# Methodological Development and Assessing Prescribing Determinants Through Cumulative Drug Exposure in Hospitalized Patients: Proof-of-Concept Retrospective Study

**DOI:** 10.2196/76961

**Published:** 2026-04-16

**Authors:** Mathilde Bories, Guillaume Bouzillé, Marc Cuggia, Pascal Le Corre, Aurélie Bannay

**Affiliations:** 1 Inserm, Laboratoire Traitement du Signal et de l’Image—UMR 1099, Centre Hospitalier Universitaire de Rennes, Université de Rennes, LTSI - Laboratoire Traitement du Signal et de l'Image, Université de Rennes, Campus Santé - Bâtiment 15b, 2 avenue du Professeur Léon Bernard, Rennes, 35000, France, 33 675438670; 2 Pôle Pharmacie, Secteur Pharmacotechnie et Onco-Pharmacie, Centre Hospitalier Universitaire de Rennes, Rennes, France; 3 Laboratoire de Biopharmacie et Pharmacie Clinique, Faculté de Pharmacie, Université de Rennes, Rennes, France; 4 Centre Hospitalier Universitaire de Rennes, Inserm, Ecole des hautes études en santé publique, Institut de recherche en santé, environnement et travail, UMR_S 1085, Université de Rennes, Rennes, France; 5 Université de Lorraine, Centre Hospitalier Régional Universitaire de Nancy, Centre national de la recherche scientifique, Inria, Laboratoire lorrain de recherche en informatique et ses applications, Nancy, France

**Keywords:** cumulative drug exposure, clinical data warehouse, polypharmacy, hyperpolypharmacy, drug interactions, potentially inappropriate medications

## Abstract

**Background:**

Preventing adverse drug reactions requires accurate monitoring of drug exposure throughout patient care. Conventional metrics, measured at admission or discharge, fail to capture the dynamic and cumulative nature of drug burden during hospitalization. Improving exposure assessment is essential to support clinical decision-making and medication safety. Clinical data warehouses (CDWs), which integrate detailed drug administration records, enable the retrospective reuse of hospital data to develop more granular and dynamic measures of in-hospital drug exposure.

**Objective:**

This exploratory proof-of-concept study aimed to introduce 2 cumulative drug exposure metrics computed from CDW: cumulative drug exposure (CDE) and cumulative drug exposure density (CDED). The study also aimed to compare these metrics with conventional metrics, primarily as a methodological development for characterizing prescribing determinants in hospitalized patients.

**Methods:**

We conducted a retrospective study using the eHOP CDW at Rennes University Hospital. Adults hospitalized for hematological malignancies were included. Four prescribing determinants were analyzed: polypharmacy (PP), hyperpolypharmacy (HPP), drug-drug interactions (DDIs), and potentially inappropriate medications (PIMs). CDE quantified the number of days each determinant was present, while CDED normalized this value to hospital length of stay. Analyses combined descriptive statistics, Spearman correlations, and factorial analysis of mixed data (FAMD).

**Results:**

Mean CDE values were 10.5 days for PP, 5.7 for HPP, 64.7 for DDIs, and 19.0 for PIMs (≥65 years). CDED values ranged from 0.3 to 3.2. Conventional metrics at admission were weakly correlated with cumulative exposure measures (eg, DDIs: *r*_s_=0.04, *P=.*752; PP: *r*_s_=–0.04, *P=*.757; HPP: *r*_s_=0.11, *P=*.364). Stronger, significant correlations emerged at discharge (CDE DDIs: *r*_s_=0.44, CDED: *r*_s_=0.46; both *P<*.001). PIMs showed strong significant correlations at both time points. FAMD highlighted that cumulative metrics contributed independently to the principal components, capturing dynamics of drug exposure not reflected by conventional indicators.

**Conclusions:**

CDE and CDED, derived from real-time CDW data, offer reproducible and scalable alternatives to conventional metrics for characterizing drug exposure in patients hospitalized for severe conditions. They provide a more accurate characterization of drug burden and hold promise for pharmacoepidemiological research and clinical decision support.

## Introduction

### Background

Preventing adverse drug reactions (ADRs) is a major challenge for health care systems worldwide, both to improve patient outcomes and to reduce economic burdens [1,2]. Monitoring drug exposure at both the patient and institutional levels is a key strategy to address this issue. Furthermore, information on how drug exposure influences patient response is fundamental to evaluating a medication’s safety and effectiveness. Therefore, various tools such as Computerized Provider Order Entry, clinical decision support systems (CDSS), and deprescribing programs have been implemented to limit unnecessary or inappropriate prescriptions [3,4]. Nonetheless, significant challenges remain: generating only relevant alerts to avoid clinician alert fatigue and providing meaningful indicators to assess the effectiveness of deprescribing programs. All of these initiatives depend on accurate assessment of patients’ drug exposure across all stages of care [5].

Learning health system (LHS) aims to address this challenge by capturing data from routine practice, transforming them into knowledge, and reintegrating that knowledge to improve care in a continuous cycle. A typical LHS includes three iterative steps:

1. *Practice-to-data*: Data are collected from routine clinical practice, primarily through electronic health records (EHRs) and integrated into appropriate data reuse architecture such as clinical data warehouse (CDW).
2. *Data-to-knowledge*: The available data are analyzed to generate new knowledge, using methods such as statistical modeling, data mining, or machine learning, to identify patterns and predictors.
3. *Knowledge-to-practice*: The newly generated knowledge is translated back into clinical practice via decision support tools, updated protocols, or quality improvement initiatives, creating a continuous feedback loop that promotes better health care delivery and patient outcomes.

Hence, LHSs could contribute to improving current practices in drug exposure measurement by enabling the development of new methods for knowledge extraction from real-world data.

### Current Knowledge and Practices on Drug Exposure Measurement

Accurately quantifying drug exposure is fundamental for assessing medication safety and effectiveness. A robust theoretical framework integrates pharmacokinetics, pharmacoepidemiology, and clinical pharmacology, with 4 dimensions: exposure definition, data sources, metrics, and biases [6]. Pharmacokinetic exposure can be described by parameters such as the area under the curve, maximum and minimum concentrations (Cmax, Cmin), or time in therapeutic range; despite being closely related to drug effects, these concentration-based measures are rarely used, even in clinical trials where the administered dose remains the standard [7]. Pharmacoepidemiology defines exposure by drug receipt and refines it with duration, dose, and adherence. Aggregate indicators such as medication possession ratio, proportion of days covered, and the World Health Organization–defined daily dose are useful in chronic care but fail to capture rapid regimen changes or cumulative burden in hospital settings.

Because inpatient treatments vary from day to day, choosing an appropriate observation window is crucial. Assessments limited to admission or discharge underestimate drug burden; a comprehensive evaluation should encompass the entire hospital stay. In addition to quantifying overall exposure, specific prescribing determinants provide complementary insight into the quantity and quality of therapy. Polypharmacy (PP) and hyperpolypharmacy (HPP) reflect the number of concomitant drugs, while potentially inappropriate medications (PIMs) and drug-drug interactions (DDIs) capture the appropriateness of the regimen. These indicators are particularly relevant for older or multimorbid patients.

Whether evaluating overall exposure or these determinants, the same methodological challenges arise. Exposure estimated from prescriptions may overstate administration, while unrecorded medications obtained elsewhere cause underestimation; differences in length of stay introduce further bias, as longer stays inevitably yield more exposure and more determinants. These issues, combined with the dynamic nature of inpatient therapy, expose the limitations of simple point-in-time indicators that record the presence of drugs or determinants at admission, during hospitalization, or at discharge.

To more faithfully characterize drug exposure, especially in contexts of transient high drug burden such as intensive care units or transplantation services, composite measures that integrate both intensity and accumulation are needed. Dose intensity (total dose or number of drugs per unit time), dose density (dosing frequency), and cumulative exposure (sum of doses over the stay) capture both the quantity and the temporal dynamics of medication use. By providing a fuller picture than conventional point-in-time metrics, these cumulative indicators can support more nuanced analyses of prescribing practices and promote safer, more rational medication use.

### Date Reuse and the LHS to Improve Measure of Drug Exposure

The reuse of health data has become an essential component of medical research, offering insights into patient care, treatment effectiveness, and disease patterns. Through the integration of EHRs, administrative databases, and insurance claims, researchers can analyze large-scale populations and track health care trends over time [8].

Among the most powerful tools facilitating the practice-to-data step of LHS are CDWs, which aggregate structured and unstructured health information from multiple sources within hospital systems [9]. By consolidating laboratory results, drug administration records, and patient demographics, CDWs provide invaluable real-world evidence for pharmacovigilance, pharmacoepidemiology, and drug safety research [10]. Moreover, unlike traditional clinical trial data, which often lack generalizability to broader populations, they provide real-world insights into prescribing practices, ADRs, and treatment effectiveness in heterogeneous patient groups [11].

Hospital-based CDWs offer a unique advantage by capturing detailed, real-time data on medication administration, making them particularly valuable for drug exposure. Unlike outpatient prescription databases, which, in most cases, indicate only intended treatments rather than actual drug intake, hospital CDWs provide precise records of administered medications, dosage adjustments, and the timing of drug exposure. Moreover, they facilitate precise evaluation of actual drug administration, offering dynamic analyses over the entire course of hospitalization [12,13].

However, despite their potential, the reuse of health data and CDW-based analyses faces several challenges. Data quality issues, such as missing or inconsistent medication records, variations in documentation practices across hospitals, and incomplete capture of drug administration details, can impact the reliability of findings [8]. Additionally, differences in exposure definitions such as whether drug exposure is measured based on prescriptions, administrations, or estimated treatment durations can lead to variability in results. These discrepancies highlight the need for harmonized methodologies to ensure consistency in developing new drug exposure, particularly in hospitalized patients where medication regimens are frequently modified. Standardized approaches to data integration, exposure quantification, and analytical techniques are crucial to improving the robustness and comparability of real-world evidence on medication use.

### Our Contribution

In this context, we introduce two novel cumulative metrics designed to provide a more comprehensive evaluation of drug exposure and prescribing determinants during hospitalization:

1. *Cumulative drug exposure* (*CDE*): It quantifies the total number of days a patient is exposed to PP, HPP, DDIs, or PIMs during hospitalization.
2. *Cumulative drug exposure density* (*CDED*): It normalizes CDE by the length of hospital stay to account for exposure intensity.

These metrics aim to offer a dynamic, patient-centered evaluation of drug exposure, addressing limitations of conventional static indicators and could serve as quality indicators in initiatives promoting safer and more rational use of medications.

The objective of this exploratory study was to develop and validate, as a methodological proof of concept, 2 CDE metrics (CDE and CDED) derived from real-time hospital CDW data. This work should be considered a preliminary methodological step rather than a definitive evaluation. The proposed new indicators were applied to quantify 4 key prescribing determinants (PP, HPP, DDIs, and PIMs) and were compared with traditional point-in-time measures to assess their added value within an LHS for characterizing drug exposure in hospitalized patients undergoing complex and dynamic treatment regimens.

## Methods

### Data Sources

The data for this study were obtained from three data sources:

1. CDW (eHOP): eHOP, developed at Rennes University Hospital, integrates administrative and clinical data from EHR [14]. It provides structured data, such as drug administration records, and unstructured data, such as clinical notes. For this study, structured data were extracted, including demographic details, drug names, dosages, routes of administration, and administration dates. These data facilitated detailed longitudinal tracking of drug use during hospitalization, which is critical for the computation of cumulative exposure metrics.
2. Theriaque: Theriaque [15] is a drug knowledge database that categorizes medications based on the Anatomical Therapeutic Chemical (ATC) classification system. It offers exhaustive information on drug indications, contraindications, and DDIs. The database categorizes DDIs into 4 severity levels: contraindicated, should be avoided, precaution of use, and to take into account. For this study, these levels were mapped into major (contraindicated, should be avoided) and moderate (precaution of use, to take into account) categories to support analysis [16].
3. GO-PIM list: To identify PIMs, the GO-PIM list [17] a validated reference for geriatric oncology patients, was used.

### Prescribing Determinants

PP and HPP were defined based on the daily number of distinct drugs administered during hospitalization. Therefore, the daily number of distinct ATC classes administered during each hospitalization day was computed using structured medication administration data from eHOP. A patient was classified as having PP if the number of ATC classes per day was ≥5, and HPP if the number of ATC classes was ≥10 [18]. Chemotherapy regimens used for transplant conditioning (ATC class L01) administered before the transplantation day were excluded from this calculation to avoid an artificial overestimation of polymedication due to high-dose chemotherapy protocols. This classification was applied daily throughout hospitalization, allowing for the assessment of PP and HPP at admission, discharge, and during the hospital stay.

A DDI refers to a clinically significant unintended modification in drug exposure or response caused by the coadministration of another drug [19]. The detection of DDIs was performed using the Theriaque database, which provides predefined lists of interacting drug pairs (term 1 and term 2) based on ATC codes. A DDI was recorded when both interacting drugs were administered on the same day. DDIs were further categorized into major and moderate severity levels. The daily number of DDIs per patient was computed, and aggregated values were calculated at admission, discharge, and across hospitalization to assess exposure variations over time.

PIMs are drugs for which the risks outweigh the expected benefits in older adult patients, due to an increased risk of ADRs, toxicity, or limited clinical effectiveness [20]. For each patient aged 65 years or older, all administered medications were cross-referenced daily with the GO-PIM ATC-based classification, and any drug listed in GO-PIM was classified as a PIM. The daily number of PIMs per patient was determined, and aggregate values were calculated at admission, discharge, and throughout hospitalization to evaluate temporal patterns of PIMs exposure.

### Conventional Metrics of Drug Exposure

To compare cumulative exposure metrics with traditional methods, conventional metrics were assessed at 2 hospitalization time points: admission and discharge. For each prescribing determinant (PP, HPP, DDIs, and PIMs), presence at admission or discharge was evaluated as a binary variable indicating whether the patient met the definition at these time points.

The number of prescribing determinants at each time point was also quantified as follows:

1. *For PP and HPP*: The total number of distinct drugs administered at admission and discharge.
2. *For DDIs*: The total number of DDIs identified at each time point.
3. *For PIMs*: The total number of PIMs administered at admission and discharge.

### New Cumulative Exposure Metrics

To quantify the overall exposure to the prescribing determinants throughout hospitalization, 2 cumulative exposure metrics were computed: CDE and cumulative exposure density. CDE was defined as the total number of days a patient was exposed to each prescribing determinant during hospitalization.

**Figure 1.**
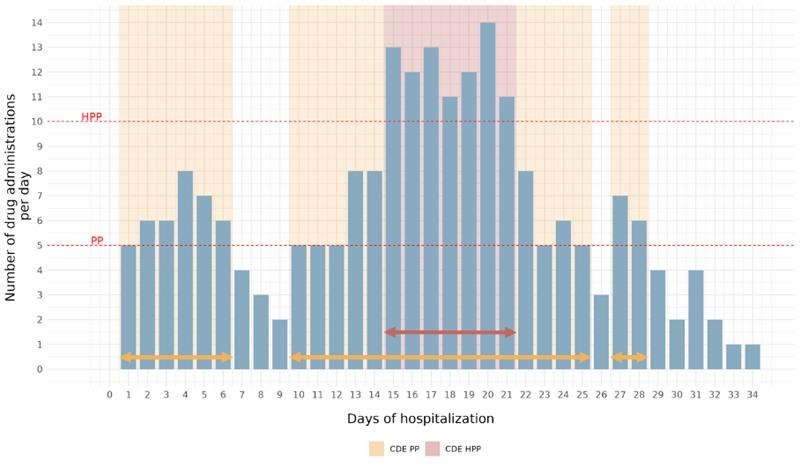
Temporal evolution of daily drug exposure and CDE in a hospitalized patient. This figure presents the daily drug exposure profile of a single patient from the study cohort over a 33-day hospitalization. The *x*-axis represents hospitalization days (days 1-34), while the *y*-axis indicates the number of distinct drugs administered per day. Thresholds for polypharmacy (PP: ≥5 drugs per day) and hyperpolypharmacy (HPP: ≥10 drugs per day) are delineated by shaded bands. CDE to PP and HPP—quantified as the total number of days the patient was exposed to these prescribing determinants—is represented by colored segments. The patient was exposed to PP from admission (day 1, 5 drugs) but was no longer exposed at discharge (day 34, 1 drug), reflecting a progressive reduction in medication burden over the hospital stay. In total, the patient had 24 days of CDE to PP (days 1-6, 10-25, and 27-28). CDE to HPP was observed for 7 days (days 15-21). This representation highlights the dynamic nature of drug exposure throughout hospitalization and underscores the limitations of static assessments at admission or discharge in capturing overall medication burden. CDE: cumulative drug exposure; HPP: hyperpolypharmacy; PP: polypharmacy.

For PP and HPP, exposure was recorded as a binary status per day (exposed and nonexposed), and the sum of exposure days was computed across the full hospital stay. However, for DDIs and PIMs, CDE accounted for the number of occurrences per day: if multiple DDIs or PIMs were recorded on a given day, each event contributed separately to the total exposure count. For instance, if a patient experienced 4 distinct DDIs on a single day, this was counted as 4 exposure days in the cumulative measure. To account for variations in hospitalization duration, CDED was calculated by normalizing CDE to the total length of stay, providing an intensity-adjusted measure (Figure 1).

### Evaluation: Use Case and Population

Hematopoietic stem cell transplantation (HSCT) patients represent a particularly high-risk group regarding ADRs, as they are exposed to an exceptionally high medication burden throughout hospitalization. Several studies have explored associations between prescribing determinants such as PP, HPP, PIMs, and DDIs, and adverse outcomes such as mortality or rehospitalization, using conventional metrics [21,22]. While these studies have highlighted potential risks, their findings remain inconsistent, likely due to methodological differences and reliance on conventional, point-in-time metrics. In most cases, drug exposure was assessed only at the time of transplantation, failing to account for the cumulative burden experienced throughout hospitalization. This limitation may lead to an incomplete assessment of medication-related risks, reinforcing the need for more dynamic and temporally sensitive approaches to exposure assessment.

HSCT treatment regimens involve prolonged use of antineoplastics, immunosuppressants, antimicrobials, and supportive therapies, making these patients especially vulnerable to PP, HPP, DDIs, and PIMs [21,23]. Given the duration and complexity of treatment, accurately quantifying CDE of prescribing determinants is essential to identify medication-related risks such as increased toxicity or adverse drug events. It could also help optimize therapeutic strategies by adjusting drug regimens and limiting unnecessary medication burden while refining prescribing guidelines through real-world evidence on exposure patterns and associated risks.

We focused on all adult patients hospitalized at Rennes University Hospital between November 1, 2020, and November 30, 2021, for autologous HSCT. Patients were identified through the diagnosis-related group of their hospital stay (ie, 27Z03) available in the Rennes University Hospital CDW, corresponding to autologous HSCT patients. Comorbidities were assessed using the Charlson Comorbidity Index, calculated from *ICD-10* (*International Statistical Classification of Diseases, Tenth Revision*) diagnostic codes available in the CDW. For patients with multiple admissions during the study period, only the first hospitalization during which the transplant procedure was performed was retained for analysis, serving as the index stay for the computation of cumulative exposure metrics.

### Statistical Analysis

#### Overview

To evaluate the relevance of cumulative exposure metrics (CDE and CDED) in reflecting overall drug exposure throughout hospitalization, statistical analyses were conducted to examine their relationship with conventional metrics at 2 specific time points: admission (first day of hospitalization) and discharge (last day of hospitalization).

The objective was to determine whether the number of medications, DDIs, and PIMs at admission and discharge, or the presence of these determinants at these time points, were correlated with cumulative exposure throughout hospitalization, or whether these conventional metrics failed to reflect the cumulative and dynamic nature of drug exposure.

The normality of the distribution of CDE, CDED, and the number of medications, DDIs, and PIMs at admission and discharge was assessed using the Shapiro-Wilk test, with a significance level of .05. A *P* value of <.05 indicated a nonnormal distribution, leading to the use of nonparametric statistical methods for subsequent analyses. All statistical analyses were performed using R (version 3.6.0; R Core Team).

#### Description of CDE and CDED Across Patients’ Groups

To describe cumulative exposure to prescribing determinants (PP, HPP, DDIs, and PIMs), patients were categorized into 4 trajectory groups based on their exposure status at admission (first day of hospitalization) and at discharge (last day of hospitalization). For each determinant, the 4 predefined trajectories were: 0→0 (not exposed at admission, nor at discharge), 0→1 (not exposed at admission but exposed at discharge), 1→0 (exposed at admission but not at discharge), and 1→1 (exposed at both admission and discharge).

For each trajectory group, descriptive statistics of cumulative exposure metrics were computed: mean, median, interquartile range, and range of both CDE and CDED. This descriptive approach aimed to characterize cumulative exposure patterns within each trajectory group, providing a detailed overview of drug burden during hospitalization.

#### Comparison of Cumulative Exposure Metrics With Conventional Metrics

To assess whether cumulative exposure throughout hospitalization was correlated with the number of administered medications, DDIs, and PIMs at admission and discharge, Spearman rank correlation coefficients were calculated. The Spearman correlation test was chosen due to the nonnormal distribution of the variables. This analysis aimed to assess the relationship between the number of medications, DDIs, and PIMs at admission and discharge with their respective CDE and CDED values, and a weak correlation (*r*_s_<0.3 [24]) would suggest that a single time point measure does not adequately reflect overall exposure, highlighting the need for cumulative exposure metrics.

In parallel, we investigated whether the presence of PP, HPP, DDIs, or PIMs at admission or discharge was associated with cumulative exposure metrics. For each determinant, the distributions of CDE and CDED were compared between patients classified as exposed versus nonexposed (eg, PP=0 vs PP=1) at both time points. Boxplots were used to visualize these distributions and examine potential differences in median cumulative exposure across groups. To support this visual comparison, the difference in median values between exposed and nonexposed groups was also calculated (*d*), providing a quantitative summary of potential disparities. This complementary, descriptive analysis provided additional insight into whether conventional binary indicators commonly used could reliably reflect cumulative drug burden in hospitalized patients.

#### Factorial Analysis of Mixed Data

To further explore the relationship between conventional metrics and cumulative exposure metrics (CDE and CDED), a factorial analysis of mixed data (FAMD) was conducted. This approach was chosen to simultaneously analyze numerical measures of drug exposure (eg, number of drugs administered and cumulative exposure) and categorical classifications of prescribing determinants (eg, presence of PP, HPP, and DDIs). The objective was to assess whether cumulative exposure metrics introduce new information beyond conventional metrics and whether they form an independent dimension in the factorial space, justifying their use as complementary or alternative measures. Two separate FAMD analyses were performed: one incorporating CDE and another focusing on CDED, each compared with conventional metrics at admission and discharge. The contribution of each variable to the principal dimensions was examined using eigenvalues and variable loadings. Variables strongly associated with a given dimension were identified to determine whether CDE or CDED formed an independent axis of variation. PIMs were excluded from the FAMD analyses as they applied exclusively to patients aged 65 years and older. Including a determinant restricted to a subgroup would have introduced structural heterogeneity and compromised the interpretability of global dimensions across the full cohort.

### Ethical Considerations

The study was conducted in accordance with the ethical principles of the 1964 Declaration of Helsinki and its amendments, or equivalent standards. Given its retrospective and noninterventional design based on existing database data, a waiver of informed consent was granted. Confidentiality and anonymity were strictly maintained throughout: the extracted data contained no nominative information and were fully deidentified prior to analysis, in compliance with the institutional data protection procedures. The protocol was approved by the local ethics committee (approval 24.12, May 2024) and complies with French clinical research regulations. The study used routinely collected hospital data. No compensation was provided to participants and no direct contact with patients occurred.

## Results

### Study Population

This study included 69 adult patients hospitalized at Rennes University Hospital between November 1, 2020, and November 30, 2021, for autologous HSCT. Patients were identified using the diagnosis-related group code (eg, 27Z03). For each patient, only the hospitalization during which the transplant procedure was performed was retained as the index stay, so that each patient contributed a single stay to the analysis. The cohort’s median age was 60 years (range 21‐70 years), with 22 patients out of 69 (31.9%) aged 65 years or older. The median length of hospital stay was 19 days (range 14‐52 days).

The primary indications for HSCT were multiple myeloma (44/69, 64%), non-Hodgkin lymphoma (19/69, 28%), and Hodgkin lymphoma (6/69, 9%). Patients exhibited a median Charlson Comorbidity Index score of 4, reflecting a significant burden of comorbid conditions.

### Prescribing Determinants

#### Overview

The analysis of prescribing determinants among hospitalized HSCT patients revealed distinct patterns in PP, HPP, DDIs, and PIMs. These results are shown in Table 1 and highlight the dynamic nature of prescribing determinants in hospitalized HSCT patients, demonstrating substantial fluctuations between admission, hospitalization, and discharge.

Nearly all patients (68/69, 99%) experienced at least 1 day of PP during hospitalization, although its prevalence was substantially lower at admission (10/69, 15%) and discharge (39/69, 58%). Similarly, HPP was present in 77% (53/69) of patients at some point during their stay but was rare at admission (1/69, 1%) and remained infrequent at discharge (1/69, 1%). DDIs were highly prevalent throughout hospitalization, with 99% (68/69) of patients experiencing at least 1 interaction, although major DDIs were less common than minor ones. At admission, no patients had a major DDI, while 36% (25/69) had a minor DDI. By discharge, 3% (2/69) of patients had major DDIs and 52% (36/69) still had minor DDIs. Although the proportion of patients with major DDIs at admission or discharge remains low, 20% (14/69) of patients were exposed to at least 1 major DDI at some point during their hospital stay. Among patients aged 65 years or older, all had at least 1 PIM during their hospitalization, with 36% (8/22) presenting with a PIM at admission and 45% (10/22) at discharge.

The mean number of drug administrations per day was 7.7 across the entire hospital stay but was lower at admission (3.0 per day) and at discharge (5.1 per day). The number of DDIs and PIMs administrations followed similar trends: 0.7 DDI per day at admission and 1.5 at discharge and 0.4 PIM per day at admission and 0.7 at discharge.

**Table 1.** Conventional metrics and cumulative drug exposure metrics for prescribing determinants during hospitalization^a^.

Variable	Hospital stay	Admission	Discharge
Presence of prescribing determinants (prevalence of patients in %)			
PP^b^	99 (68/69)	15 (10/69)	58 (39/69)
HPP^c^	77 (53/69)	1 (1/69)	1 (2/69)
DDIs^d^	99 (68/69)	36 (25/69)	52 (36/69)
DDI major	20 (14/69)	0 (0/69)	3 (2/69)
DDI minor	99 (68/69)	36 (25/69)	52 (36/69)
PIMs^e^	100 (22/22)	36 (8/22)	45 (10/22)
Number of administrations per day (mean)			
Drugs administration	7.7	3.0	5.1
DDI administration	3.2	0.7	1.5
Major	0.1	0.0	0.0
Minor	3.2	0.7	1.5
PIM administration	1.2	0.4	0.7
CDE^f^ during hospital stay (days), mean			
PP	16.3	—^g^	—
HPP	5.8	—	—
DDIs	64.7	—	—
Major	2.0	—	—
Minor	62.7	—	—
PIMs	19	—	—
CDED^h^, mean			
PP	0.8	—	—
HPP	0.3	—	—
DDIs	3.2	—	—
Major	0.1	—	—
Minor	3.1	—	—
PIMs	1.2	—	—

a This table presents the conventional metrics and cumulative drug exposure metrics for prescribing determinants, including polypharmacy (PP), hyperpolypharmacy (HPP), drug-drug interactions (DDIs), and potentially inappropriate medications (PIMs), at 3 key hospitalization time points: admission, discharge, and throughout the hospital stay. The conventional metrics include the presence of each prescribing determinant at admission and discharge, expressed as the percentage of patients who had the determinant at that specific time point. For the presence of prescribing determinants throughout the hospital stay, a patient was considered exposed if they had at least one occurrence of the determinant at any time during hospitalization. Additionally, the number of administrations at admission and discharge is reported. For PP and HPP, this corresponds to the total number of distinct medications administered per day, while for DDIs and PIMs, it represents the total number of interactions or inappropriate medications recorded at each time point. The cumulative drug exposure metrics include cumulative drug exposure (CDE), which quantifies the total number of days a patient was exposed to a prescribing determinant during hospitalization. For DDIs and PIMs, this metric accounts for multiple occurrences per day. Cumulative drug exposure density normalizes CDE by the total length of the hospital stay, providing an intensity-adjusted measure of exposure. The analysis of PIM exposure is restricted to patients aged 65 years and older.

b PP: polypharmacy.

c HPP: hyperpolypharmacy.

d DDIs: drug-drug interactions.

e PIMs: potentially inappropriate medications.

f CDE: cumulative drug exposure.

g Not available.

h CDED: cumulative drug exposure density.

#### Comparison of Cumulative Exposure Metrics Across Patient Groups

To further describe CDE across prescribing determinant trajectories (PP, HPP, DDIs, and PIMs), detailed descriptive statistics were calculated for both CDE and CDED, including mean, median (IQR), and range. Patients classified as exposed at both admission and discharge (1→1) generally showed the highest CDE and CDED values for each prescribing determinant, indicating sustained exposure throughout hospitalization. For example, in this group, median CDE reached 17.5 days (14.5‐19.8) for PP and 60 days (51-132) for DDIs, corresponding to median CDED values close to 1.0 and 3.5, respectively, indicating sustained exposure throughout hospitalization. However, nonnegligible cumulative exposure was also observed in 0→1 trajectories, particularly for DDIs where the median CDE was 69 days (31-122), highlighting the limitations of conventional point-in-time metrics in capturing dynamic exposure patterns. Even among 0→0 patients, who were not exposed at either time point, cumulative exposure levels remained relatively high. For example, the median CDE was 14 days (10-18) for PP and 31 days (18-63) for DDIs, suggesting that conventional prevalence-based indicators may underestimate transient or intermediate exposure. Full descriptive results are provided in Multimedia Appendix 1.

#### Comparison of Cumulative Exposure Metrics With Conventional Metrics

**Figure 2.**
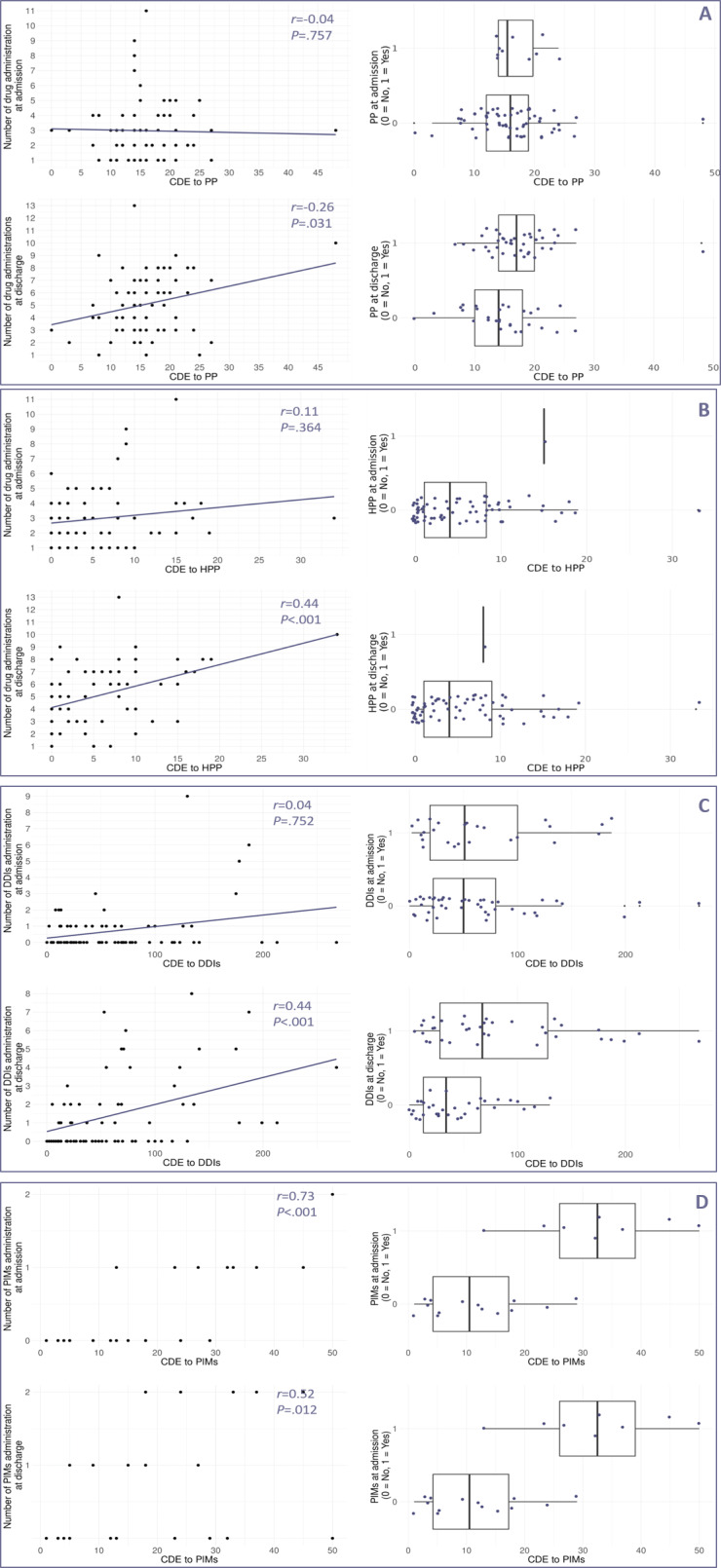
Comparison of conventional metrics with CDE across prescribing determinants (PP, HPP, DDIs, and PIMs). This figure examines the relationship between conventional metrics at admission and discharge and CDE over the entire hospitalization for each prescribing determinant: PP, HPP, DDIs, and PIMs. Each panel (A, B, C, and D) corresponds to a specific prescribing determinant: (A) PP, (B) HPP, (C) DDIs, and (D) PIMs. For each prescribing determinant, 2 types of analyses were conducted: (1) Correlation analysis (scatter plots, left side): the association between the number of administrations at admission (top left) and discharge (bottom left) and CDE was assessed using Spearman rank correlation coefficient (*r*) and regression lines. (2) Comparison of distributions (boxplots, right side): the exposure status of patients to each prescribing determinant (binary: 0 = not exposed, 1 = exposed) at admission (top right) and discharge (bottom right) was compared against CDE using boxplots. Differences in CDE were evaluated descriptively by examining the distribution and median CDE values between exposed and nonexposed patients. A weak correlation (*r*s <0.3) or minimal differences in CDE distributions between exposed and nonexposed patients at admission or discharge suggest that a single time point measure may not adequately reflect CDE, emphasizing the need for longitudinal assessment. Conversely, stronger correlation (*r*s close to 1) or pronounced distributional differences suggests that conventional metrics at admission or discharge may serve as reliable proxies for CDE. CDE: cumulative drug exposure; DDIs: drug-drug interactions; HPP: hyperpolypharmacy; PIMs: potentially inappropriate medications; PP: polypharmacy.

Correlation and distribution analyses highlighted heterogeneous relationships between conventional metrics and cumulative exposure metrics (Figures2  3).

For PP, HPP, and DDIs, correlations with CDE and CDED at admission were weak and nonsignificant (*r*_s_<0.3 − *P>*.05), while stronger significant associations emerged at discharge for PP (*r*_s_CDED=0.43 − *P*<.001), HPP (*r*_s_CDE=0.44 − *P*<.001; *r*_s_CDED=0.46 − *P*<.001) and DDIs (*r*_s_ CDE=0.44 − *P*<.001; *r*_s_ CDED=0.46 − *P*<.001).

In contrast, PIMs showed consistently strong correlations with both CDE and CDED at admission and discharge (*r*_s_ CDE=0.73 − *P*<.001, *r*_s_ CDED=0.71 − *P*<.001 at admission; and *r*_s_ CDE=0.52 − *P=*.012, *r*_s_ CDED=0.59 − *P*=.004 at discharge). Due to the discrete nature of PIM administration (maximum of 1 or 2 drugs at admission or discharge), regression lines were not plotted for PIMs in the scatterplots, as they did not meaningfully represent trends in the data.

Distribution comparisons (boxplots) further confirmed that the presence of PP or DDIs at admission was not associated with noticeably higher cumulative exposure. In contrast, PIM presence at admission consistently corresponded to increased CDE and CDED values. At discharge, only DDIs and PIMs were visibly associated with higher cumulative exposure, while differences for PP remained limited. All results are summarized in Multimedia Appendix 2.

**Figure 3.**
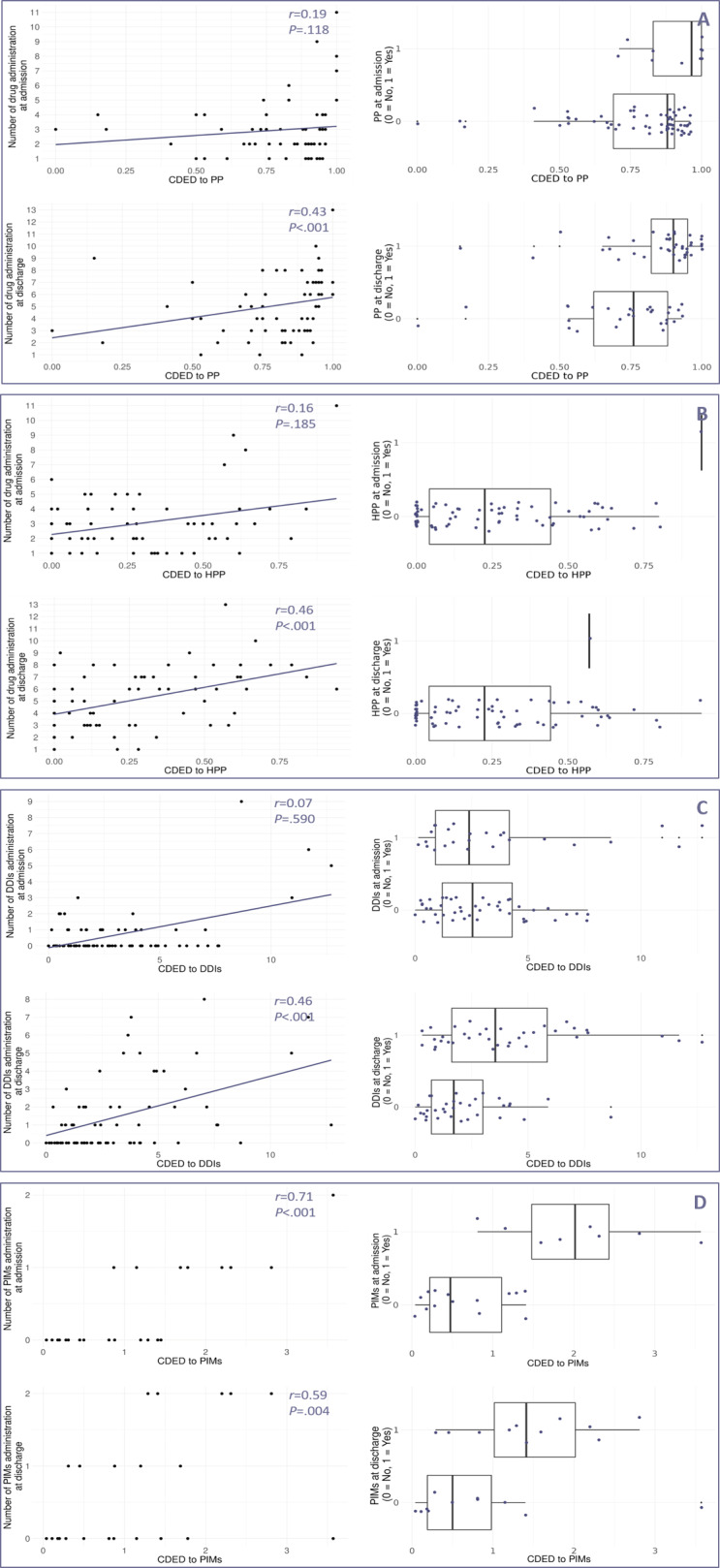
Comparison of conventional metrics with CDED across prescribing determinants (PP, HPP, DDIs, and PIMs). This figure examines the relationship between conventional metrics at admission and discharge and CDED over hospitalization for each prescribing determinant: PP, HPP, DDIs, and PIMs. Each panel (A, B, C, and D) corresponds to a specific prescribing determinant: (A) PP, (B) HPP, (C) DDIs, and (D) PIMs. For each prescribing determinant, two types of analyses were conducted. (1) Correlation analysis (scatter plots, left side): the association between the number of administrations at admission (top left) and discharge (bottom left), and CDED was assessed using Spearman rank correlation coefficient (*r*) and regression lines. (2) Comparison of distributions (boxplots, right side): the exposure status of patients to each prescribing determinant (binary: 0 = not exposed, 1 = exposed) at admission (top right) and discharge (bottom right) was compared against CDED using boxplots. Differences in CDED were evaluated descriptively by examining the distribution and median CDED values between exposed and nonexposed patients. A weak correlation (*r*s<0.3) or minimal differences in CDED distributions between exposed and nonexposed patients at admission or discharge suggest that a single time point measure may not adequately reflect CDED, emphasizing the need for longitudinal assessment. Conversely, stronger correlation (*r*s close to 1) or pronounced distributional differences suggest that conventional metrics at admission or discharge may serve as reliable proxies for CDED. CDED: cumulative drug exposure density; DDIs: drug-drug interactions; HPP: hyperpolypharmacy; PIMs: potentially inappropriate medications; PP: polypharmacy.

#### Factorial Analysis of Mixed Data

**Figure 4.**
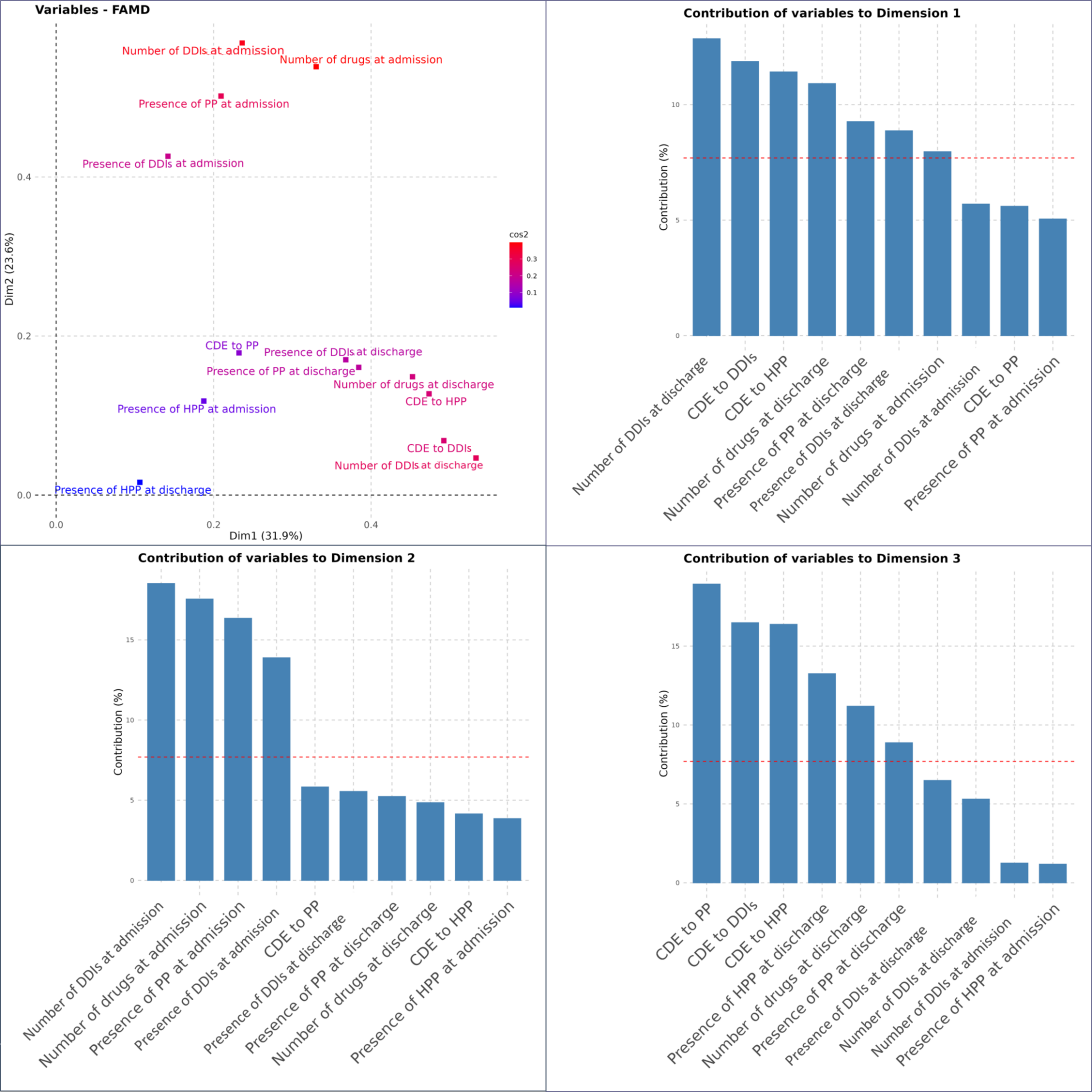
Representation and contribution of variables in the FAMD on CDE and conventional metrics. This figure presents the results of the FAMD conducted to compare CDE metrics with conventional drug metrics. The top-left panel illustrates the factor space representation of variables, where the color gradient reflects the cos² values, indicating the quality of representation in the first 2 dimensions. The 3 bar plots display the contribution (%) of each variable to the formation of the first 3 principal dimensions in the FAMD performed to compare CDE with conventional drug exposure metrics. Each panel represents 1 of the 3 dimensions. The red dashed line represents the expected average contribution threshold, indicating variables that contribute more than expected to each dimension. CDE: cumulative drug exposure; DDIs: drug-drug interactions; FAMD: factorial analysis of mixed data; HPP: hyperpolypharmacy; PP: polypharmacy.

To assess how cumulative exposure measures relate to conventional drug exposure metrics, 2 separate FAMD analyses were conducted: one incorporating CDE and another integrating CDED. Full details, including variable contributions and graphical representations, are provided in Figures4  5 and in MultimediaAppendices 3  4.

The FAMD on CDE metrics and conventional metrics shown in Figure 4 identified 3 principal dimensions, explaining 69.2% of the total variance (31.9% for dimension 1, 23.6% for dimension 2, and 13.7% for dimension 3). The first and third dimensions present a mixed structure, integrating both conventional metrics (eg, number of drugs and DDIs at admission and discharge, presence of PP or HPP) and cumulative exposure metrics (CDE to DDIs and HPP for dimension 1; CDE to PP and CDE to DDIs and HPP for dimension 3). In contrast, the second dimension is shaped almost exclusively by conventional metrics, particularly variables describing the patient’s medication profile at admission.

**Figure 5.**
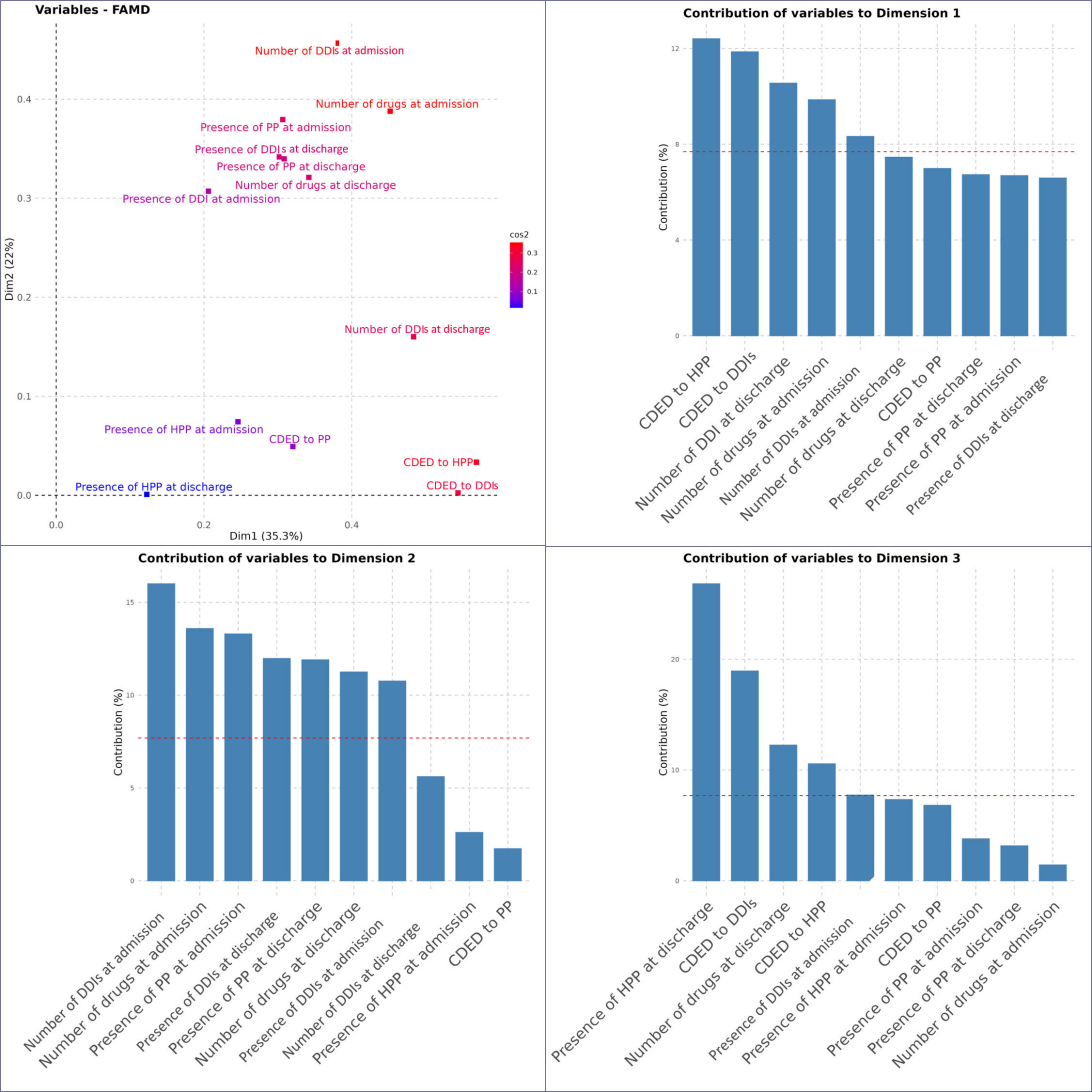
Representation and contribution of variables in the FAMD on CDED and conventional metrics. This figure presents the results of the FAMD conducted to compare CDED metrics with conventional drug metrics. The top-left panel illustrates the factor space representation of variables, where the color gradient reflects the cos² values, indicating the quality of representation in the first 2 dimensions. The 3 bar plots display the contribution (%) of each variable to the formation of the first 3 principal dimensions in the FAMD performed to compare CDED with conventional metrics. Each panel represents 1 of the 3 dimensions. The red dashed line represents the expected average contribution threshold, indicating variables that contribute more than expected to each dimension. CDED: cumulative drug exposure density; DDIs: drug-drug interactions; FAMD: factorial analysis of mixed data; HPP: hyperpolypharmacy; PP: polypharmacy.

The FAMD on CDED metrics and conventional metrics presented in Figure 5 identified 3 principal dimensions, explaining 68.3% of the total variance (35.3% for dimension 1, 21.9% for dimension 2, and 11.1% for dimension 3).

Dimension 1 was mainly driven by CDED to HPP and CDED to DDI metrics, alongside conventional metrics such as the number of medications at admission and discharge, as well as the number of DDIs at both time points. This suggests that dimension 1 integrates both conventional metrics and CDE metrics. Dimension 2, by contrast, was solely structured by conventional metrics, with no significant contribution from CDED variables. Dimension 3 reflected a combination of conventional metrics (presence of HPP at discharge and number of drugs at discharge) and CDED metrics related to HPP and DDIs further supporting the complementary role of CDED metrics in capturing dense and variable drug exposure patterns.

## Discussion

### Principal Results

This exploratory study aimed to develop and evaluate 2 CDE metrics: CDE and CDED, and to compare them to conventional, point-in-time indicators in a cohort of autologous HSCT recipients. These metrics were designed to capture the number of days during which patients were exposed to PP, HPP, DDIs, and PIMs, with CDED normalizing this duration by length of stay. Compared with binary measures at admission or discharge, CDE and CDED offered a more nuanced picture of drug burden by incorporating treatment duration and intensity.

Across all prescribing determinants, the number of drugs and DDIs at admission showed no significant correlation with CDE or CDED, and the presence of PP, HPP, or DDIs at admission was not associated with higher CDE or CDED values. These findings suggest that admission-based conventional metrics do not reliably reflect the overall drug burden experienced by patients throughout hospitalization. Conversely, while the number of medications at discharge was not correlated with cumulative exposure to PP, a moderate correlation was observed with cumulative exposure to HPP. Similarly, the number of DDIs at discharge showed a significant positive correlation with cumulative exposure to DDIs.

These findings can be explained by the specific therapeutic trajectory of autologous HSCT. Drug exposure evolves dynamically throughout the hospital stay; it is not determined solely by the patient’s status at admission or discharge but by successive clinical phases such as conditioning, aplasia, and management of complications or auto-graft versus host disease [25-27]. Numerous medications (antimicrobials, antivirals, antifungals, immunosuppressants, and supportive care drugs) are initiated to prevent or manage infections, mucositis, nausea, and hematologic toxicities, and many are maintained until discharge and sometimes beyond [23,28]. This progressive accumulation of treatments contributes to cumulative exposure and helps explain why discharge metrics better correlate with cumulative measures.

The heterogeneity of associations reported in the literature between conventional metrics and clinical outcomes in malignant hematological diseases further underscores the limitations of static indicators. Studies have linked PP, PIMs, and DDIs to increased toxicity, reduced survival, or poorer transplantation outcomes, but the strength and consistency of these associations vary widely depending on definitions, study populations, (autologous vs allogeneic HSCT), and the timing of measurement [21,22,29,30]. Such divergences highlight the need for dynamic and integrative exposure measures that better reflect the complexity of pharmacological trajectories.

To further explore whether cumulative metrics capture distinct aspects of drug exposure, we conducted an FAMD. When CDE variables were included, the principal components were largely driven by conventional metrics such as the number of medications and the presence of PP or DDIs at admission and discharge; CDE variables were less strongly represented, suggesting that they capture specific dimensions not fully reflected by static measures. In contrast, when CDED was included, this exposure density measure contributed more substantially to the first dimensions, alongside some conventional metrics, indicating that normalizing cumulative exposure by length of stay captures relevant contrasts between patients.

PIMs were not incorporated into the FAMD because they apply only to patients aged  65 years and older, and including a determinant restricted to a subpopulation would have introduced structural heterogeneity. The dynamics of PIM exposure, often linked to chronic or preexisting treatments, differ from those of other determinants: conventional PIM metrics tend to align closely with cumulative measures because these medications are commonly part of long-term regimens started before hospitalization and continued during and after the inpatient period [31,32].

### Limitations

This study has several limitations. First, the relatively small sample size (n=69) and the single-center design inevitably limit the statistical power of the analyses and the generalizability of the findings. Accordingly, this work should be interpreted primarily as an exploratory methodological development rather than as a definitive evaluation of drug exposure in HSCT patients. The study population consisted exclusively of autologous HSCT recipients, a highly specific group treated under standardized therapeutic protocols. This deliberate choice was made because these patients are exposed to dense and complex drug burden, providing a relevant framework to test and refine the proposed metrics. However, the conceptual approach underlying CDE and CDED is not restricted to autologous HSCT recipients: these cumulative measures are designed to be clinically agnostic and can be applied to other hospitalized populations, regardless of their pharmacological profile. While their relevance may be particularly evident in contexts of high therapeutic intensity such as oncology, intensive care, or surgery, they are generalizable to any setting where drug exposure evolves dynamically over time. Future work should therefore focus on validating these metrics in larger, multicenter, and more diverse clinical populations, ideally including outcome analyses to establish their clinical significance.

Second, clinical outcomes such as toxicity, adverse drug events, or hospital readmission were not assessed in this study. While this limits the immediate clinical applicability and interpretation of cumulative exposure, the primary purpose of this work was methodological, aiming to develop and illustrate novel exposure metrics. However, ongoing work is being conducted to evaluate the relationship between these metrics and patient outcomes, in order to support their prognostic and practical value.

Third, although the use of a CDW represents a key strength by offering precise, structured information on actual drug administrations, it is not without limitations. In particular, CDWs do not systematically capture clinical indications, prescriber rationale, or therapeutic intent in a structured format. Retrieving such information would require manual review of unstructured clinical documents such as discharge summaries, without guarantee of consistency or completeness. This limitation can complicate the interpretation of the clinical appropriateness of certain prescribing patterns such as PP, HPP, or DDIs, which may, in some cases, be justified depending on the clinical context, severity of illness, or treatment objectives. Furthermore, certain contextual elements such as patient-brought medications or off-protocol adaptations may not be consistently documented in structured data fields.

### Comparison With Prior Work

The cumulative nature of drug exposure, although conceptually aligned with long-standing practices in public health, remains underexplored in pharmacoepidemiology. Established exposure metrics such as pack-years in tobacco research or cumulative indices in occupational health have long recognized the importance of duration and timing. Conceptually, this approach aligns with the broader exposome framework, which aims to characterize the totality of environmental, biological, and behavioral exposures an individual encounters across the life course [33].

Within this framework, pharmaceuticals represent a major, yet insufficiently characterized component of the chemical exposome, particularly regarding their longitudinal accumulation. Although medications are intentionally administered, their cumulative impact, especially in contexts of PP and prolonged or intensive treatment, can significantly modulate health outcomes beyond the intended therapeutic effect [34].

Recent methodological developments have sought to better account for the temporal dimension of drug exposure. Among them, the weighted cumulative exposure model has extended time-sensitive modeling to pharmacological exposures by estimating the effect of prior doses using flexible, spline-based weight functions over specified exposure windows [35]. However, these approaches rely predominantly on prescription or dispensing data and are implemented within regression-based analytic frameworks, requiring prior assumptions about exposure timing and pharmacological latency. While such models offer valuable insights for studying long-term drug effects in ambulatory care, they are less suited to settings where drug exposure is highly dynamic and context dependent. In this context, CDE and CDED leverage real-time administration data recorded in hospital CDWs, enabling standardized and reproducible quantification of actual in-hospital drug burden.

At the population level, CDE and CDED offer valuable tools to inform hospital-wide strategies for medication management. By providing standardized, temporally resolved indicators of drug exposure, they could be integrated into institutional dashboards to monitor prescribing practices across wards or clinical services. For example, identifying units with persistently high cumulative exposure to PP may help guide resource allocation, staff training, or targeted medication review initiatives. Beyond real-time monitoring, these metrics also allow for longitudinal tracking of drug exposure trends, making them particularly relevant for evaluating the impact of deprescribing policies or institutional changes in prescribing practices. Such applications directly respond to limitations identified in previous work, which highlighted that commonly used point-in-time indicators fail to adequately capture the dynamic nature of drug exposure during hospitalization and emphasized the need for cumulative, temporally informed metrics better suited to quality monitoring and prescribing optimization [36-38].

At the individual level, these new metrics could enhance both the specificity and the clinical relevance of decision support systems. Most CDSS currently rely on static medication lists and rule-based logic applied at fixed points in time, often ignoring how long or how intensively a patient has been exposed to potentially risky prescriptions. This contributes to high rates of nonspecific alerts and clinician alert fatigue [39,40].

By incorporating cumulative exposure into alerting mechanisms, CDSS could move beyond simple presence or absence logic to integrate the duration and intensity of drug exposure, thereby identifying patients with a sustained drug burden more accurately. For example, alerts could be designed to trigger only when exposure to high-risk interactions or inappropriate medications exceeds a predefined cumulative threshold, supporting more clinically meaningful interventions and reducing unnecessary interruptions. Embedding these indicators into patient-level dashboards within EHRs would also provide clinicians with a longitudinal view of drug burden at the bedside, directly informing prescribing and deprescribing decisions.

However, the definition of clinically validated cumulative exposure thresholds remains to be established. Demonstrating associations between these thresholds and meaningful patient outcomes is a critical next step, beyond the methodological proof-of-concept presented here, and would provide the necessary foundation for their integration into CDSS. This represents a relevant direction for future research, particularly for refining risk stratification and alert prioritization within digital clinical tools.

In summary, CDE and CDED address key limitations of existing exposure indicators by leveraging real-world, time-resolved data. Their integration into both institutional governance frameworks and clinical decision tools opens new perspectives for optimizing pharmacotherapy and promoting safer, more appropriate medication use in hospital settings.

### Conclusions

This study proposes 2 cumulative exposure metrics, CDE and CDED, derived from daily drug administration data recorded in hospital CDWs. By incorporating the temporal dimension of treatment, these metrics offer a more comprehensive assessment of in-hospital drug exposure than conventional point-in-time metrics. Their reproducibility and adaptability support applications ranging from research to quality monitoring and medication safety. Further analyses are underway to examine how these cumulative profiles relate to patient trajectories and clinical events and to explore their potential contribution to hospital surveillance and prescribing evaluation efforts.

## Supplementary material

10.2196/76961 Multimedia Appendix 1 Descriptive analysis of cumulative drug exposure (CDE) and cumulative drug exposure density (CDED) across prescribing determinant trajectories. This table shows descriptive statistics for CDE and CDED across patient groups categorized by their prescribing determinant trajectories: polypharmacy (PP), hyperpolypharmacy (HPP), drug-drug interactions (DDIs), and potentially inappropriate medications (PIMs). Each prescribing determinant was assessed at 2 time points: admission (first day of hospitalization) and discharge (last day of hospitalization). Based on their exposure status at these time points, patients were classified into four trajectory groups: (1) 0→0: Patients not exposed to the determinant at admission nor discharge. (2) 0→1: Patients not exposed to the determinant at admission but exposed at discharge. (3) 1→0: Patients exposed to the determinant at admission but no longer exposed at discharge. (4) 1→1: Patients exposed to the determinant at both admission and discharge. CDE represents the total number of days a patient was exposed to the prescribing determinant throughout the hospital stay (eg, number of days with polypharmacy). CDED corresponds to the ratio of CDE to the length of stay, reflecting the average intensity of exposure over time. For each trajectory group and each prescribing determinant, the table reports the number of patients on each group (n), mean, median with interquartile range (IQR), and minimum-maximum values for both CDE and CDED.

10.2196/76961 Multimedia Appendix 2 Correlation and distribution comparisons between conventional metrics and cumulative drug exposure metrics. This table shows the results of Spearman correlation assessing the relationship between conventional metrics at admission and discharge and cumulative drug exposure (CDE) and cumulative drug exposure density (CDED) for each prescribing determinant: polypharmacy (PP), hyperpolypharmacy (HPP), drug-drug interactions (DDIs), and potentially inappropriate medications (PIMs). Table A corresponds to CDE, quantifying total exposure days. Table B corresponds to CDED, which normalizes exposure to hospital stay length. Spearman rank correlation (*r*ₛ) assessed the relationship between the number of drug administrations at admission or discharge and CDE or CDED. A strong correlation (*r*ₛ close to 1 or −1) suggests that the number of administrations at a single time point is predictive of cumulative exposure. The difference in median CDE or CDED values between exposed (1) and nonexposed (0) patients provides a complementary perspective on the extent to which conventional, binary time point metrics reflect overall cumulative drug burden.

10.2196/76961 Multimedia Appendix 3 Representation and contribution of variables in the factorial analysis of mixed data (FAMD) performed on cumulative drug exposure (CDE) and conventional metrics. This table shows the results of the FAMD performed to compare CDE with conventional metrics, including the number of drug administrations, the number of drug interactions, and the presence of prescribing determinants—polypharmacy (PP), hyperpolypharmacy (HPP), and drug-drug interactions (DDIs)—at admission and discharge. For each variable, two parameters are provided across the first 3 principal dimensions. (1) Squared cosine (cos²): It indicates the quality of representation of a variable on a given dimension. Higher values (closer to 1) suggest strong alignment with that dimension, while lower values indicate dispersion across multiple dimensions. (2) Contribution (%): It represents the extent to which a variable influences the formation of a given dimension. Variables with higher contributions play a more prominent role in defining the corresponding factorial axis.

10.2196/76961 Multimedia Appendix 4 Representation and contribution of variables in the factorial analysis of mixed data (FAMD) performed on cumulative drug exposure density (CDED) and conventional metrics. This table shows the results of the FAMD performed to compare CDED with conventional drug exposure metrics, including the number of drug administrations, the number of drug interactions, and the presence of prescribing determinants—polypharmacy (PP), hyperpolypharmacy (HPP), and drug-drug interactions (DDIs)—at admission and discharge. For each variable, two parameters are provided across the first 3 principal dimensions. (1) Squared cosine (cos²): It indicates the quality of representation of a variable on a given dimension. Higher values (closer to 1) suggest strong alignment with that dimension, while lower values indicate dispersion across multiple dimensions. (2) Contribution (%): It represents the extent to which a variable influences the formation of a given dimension. Variables with higher contributions play a more prominent role in defining the corresponding factorial axis.

## Abbreviations

ADR: adverse drug reaction
ATC: Anatomical Therapeutic Chemical
CDE: cumulative drug exposure
CDED: cumulative drug exposure density
CDSS: clinical decision support system
CDW: clinical data warehouse
DDI: drug-drug interaction
EHR: electronic health record
FAMD: factorial analysis of mixed data
HPP: hyperpolypharmacy
HSCT: hematopoietic stem cell transplant
*ICD-10*: *International Statistical Classification of Diseases, Tenth Revision*
LHS: learning health system
PIM: potentially inappropriate medication
PP: polypharmacy
