# Supplementary material for: Methodological Development and Assessing Prescribing Determinants Through Cumulative Drug Exposure in Hospitalized Patients: Proof-of-Concept Retrospective Study
Source: JMIR Med Inform. 2026 Apr 16;14:e76961. doi: 10.2196/76961 (PMC13086184; doi:10.2196/76961)
Supplement: Multimedia Appendix 1 [file medinform-v14-e76961-s001.docx]

|  | **n** | **CDE** | | | **CDED** | | |
| --- | --- | --- | --- | --- | --- | --- | --- |
|  |  | Mean | Median [IQR] | Min-max | Mean | Median [IQR] | Min-max |
| **PP** |  |  |  |  |  |  |  |
| 0 – 0 | 25 | 14.2 | 14 [10 – 18] | 0 - 27 | 0.7 | 0.8 [0.6 – 0.9] | 0 - 0.9 |
| 0 – 1 | 34 | 17.6 | 17 [14 – 19.8] | 7 - 48 | 0.8 | 0.9 [0.8 – 0.9] | 0.2 - 1 |
| 1 – 0 | 4 | 16.8 | 14.5 [14 – 17.2] | 14 – 24 | 0.8 | 0.8 [0.7 – 0.9] | 0.7 - 0.9 |
| 1 – 1 | 6 | 17.3 | 17.5 [14.5 – 19.8] | 14 – 21 | 1 | 1 [1 – 1] | 0.8 - 1 |
| **HPP** |  |  |  |  |  |  |  |
| 0 – 0 | 67 | 5.6 | 4 [1 – 8.5] | 0 – 33 | 0.3 | 0.2 [0 – 0.8] | 0 – 0.8 |
| 0 – 1 | 1 | 8.0 | 8 [8 – 8] | 8 – 8 | 0.6 | 0.6 [0.6 – 0.6] | 0.6 – 0.6 |
| 1 – 0 | 1 | 15.0 | 15 [15 – 15] | 15 – 15 | 0.9 | 0.9 [0.9 – 0.9] | 0.9 – 0.9 |
| **DDI** |  |  |  |  |  |  |  |
| 0 – 0 | 21 | 39.9 | 31 [18 – 63] | 0 – 116 | 1.9 | 1.7 [0.9 – 2.6] | 0 – 4.8 |
| 0 – 1 | 23 | 86.5 | 69 [31 – 122] | 4 – 280 | 3.8 | 4 [1.7 – 5.7] | 0.3 – 7.6 |
| 1 – 0 | 12 | 43.3 | 31 [12 – 60] | 2 – 130 | 2.2 | 1.5 [0.6 – 2.7] | 0.1 – 8.7 |
| 1 – 1 | 13 | 87.4 | 60 [51 - 132] | 13 – 187 | 5.0 | 3.5 [2.3 – 6.6] | 0.9 – 12.7 |
| **PIM** |  |  |  |  |  |  |  |
| 0 – 0 | 8 | 8.8 | 4.5 [3 – 12.2] | 1 – 29 | 0.4 | 0.2 [0.2 – 0.6] | 0 – 1.4 |
| 0 – 1 | 6 | 14.8 | 16.5 [10.5 – 18] | 5 – 23 | 0.9 | 1 [0.5 – 1.3] | 0.3 – 1.4 |
| 1 – 0 | 3 | 28.7 | 23 [18 – 36.5] | 13 – 50 | 1.8 | 1.1 [1.0 – 2.4] | 0.8 – 3.6 |
| 1 – 1 | 5 | 35.0 | 35 [33 – 37] | 27 – 45 | 2.1 | 2.2 [1.8 – 2.3] | 1.6 – 2.8 |

**Supplementary data 1: Descriptive analysis of Cumulative Drug Exposure (CDE) and Cumulative Drug Exposure Density (CDED) across prescribing determinant trajectories.**

This table presents descriptive statistics for cumulative drug exposure (CDE) and cumulative drug exposure density (CDED) across patient groups categorized by their prescribing determinant trajectories: polypharmacy (PP), hyperpolypharmacy (HPP), drug-drug interactions (DDI), and potentially inappropriate medications (PIM). Each prescribing determinant was assessed at two timepoints: admission (first day of hospitalization) and discharge (last day of hospitalization).

Based on their exposure status at these timepoints, patients were classified into four trajectory groups:

- 0→0: Patients not exposed to the determinant at admission nor discharge.

- 0→1: Patients not exposed to the determinant at admission but exposed at discharge. .

- 1→0: Patients exposed to the determinant at admission but no longer exposed at discharge.

- 1→1: Patients exposed to the determinant at both admission and discharge.

CDE represents the total number of days a patient was exposed to the prescribing determinant throughout the hospital stay (e.g., number of days with polypharmacy). CDED corresponds to the ratio of CDE to the length of stay, reflecting the average intensity of exposure over time. For each trajectory group and each prescribing determinant, the table reports the number of patients on each group (n), mean, median with interquartile range [IQR], and minimum–maximum values for both CDE and CDED
