# Supplementary material for: Methodological Development and Assessing Prescribing Determinants Through Cumulative Drug Exposure in Hospitalized Patients: Proof-of-Concept Retrospective Study
Source: JMIR Med Inform. 2026 Apr 16;14:e76961. doi: 10.2196/76961 (PMC13086184; doi:10.2196/76961)
Supplement: Multimedia Appendix 2 [file medinform-v14-e76961-s002.docx]

| **A** | **CDE - Number of administrations**  **(*Spearman – 95IC)*** | **Difference in median CDE (**d**) between exposed (1) and non-exposed (0) patients** |
| --- | --- | --- |
| **Admission** |  | |
| PP | r_s_ = -0.04  [-0.20 ; 0.20]  *( P = .757)* | d = 4 (14 – 10) |
| HPP | r_s_ = 0.11  [-0.12 ; 0.32]  *(P = .364)* | d = 11 (15 – 4) |
| DDI | r_s_ = 0.04  [-0.21 ; 0.28]  *(P = .752)* | d = -0.5 (51 – 51.5) |
| PIM | r_s_ = 0.73  [0.44 ; 0.88]  *(P < .001)* | d = 22.5 (33 – 10.5) |
| **Discharge** |  | |
| PP | r_s_ = 0.26  [-0.03 ; 0.46]  *(P = .066)* | d = -0.5 (10 – 10.5) |
| HPP | r_s_ = 0.44  [0.20 ; 0.61]  *(P < .001)* | d = 4 (8 – 4) |
| DDI | r_s_ = 0.44  [0.18 ; 0.58]  *(P < .001)* | d = 37.5 (68.5 – 31) |
| PIM | r_s_ = 0.52  [0.04 ; 0.78]  *(P = .03)* | d = 12 (24 – 12) |
|  | | |
| **B** | **CDED - Number of administrations**  **(*Spearman)*** | **Difference in median CDED (**d**) between exposed (1) and non-exposed (0) patients** |
| **Admission** |  |  |
| PP | r_s_ = 0.19  [-0.06 ; 0.43]  *(P = .118)* | d = 0.16 (0.70 – 0.54) |
| HPP | r_s_ = 0.16  [-0.09 ; 0.38]  *(P = .185)* | d = 0.71 (0.94 – 0.23) |
| DDI | r_s_ = 0.07  [-0.21 ; 0.32]  *(P = .590)* | d = -0.04 (2.33 – 2.37) |
| PIM | r_s_ = 0.71  [0.43 ; 0.88]  *(P < .001)* | d = 1.54 (2.02 – 0.48) |
| **Discharge** |  | |
| PP | r_s_ = 0.43  [0.25 ; 0.63]  *(P = <.001)* | d = -0.04 (0.52 – 0.56) |
| HPP | r_s_ = 0.46  [0.24 ; 0.63]  *(P < .001)* | d = 0.34 (0.57 – 0.23) |
| DDI | r_s_ = 0.46  [0.21 ; 0.60]  *(P < .001)* | d = 1.93 (3.62 – 1.69) |
| PIM | r_s_ = 0.59  [0.08 ; 0.81]  *(P = .004)* | d = 0.91 (1.41 – 0.50) |

**Supplementary data 2: Correlation and distribution comparisons between conventional metrics and cumulative drug exposure metrics.** This table presents the results of Spearman correlation assessing the relationship between conventional metrics at admission and discharge and cumulative drug exposure (CDE) and cumulative drug exposure density (CDED) for each prescribing determinant: polypharmacy (PP), hyperpolypharmacy (HPP), drug-drug interactions (DDI), and potentially inappropriate medications (PIM).

Table A corresponds to CDE, quantifying total exposure days. Table B corresponds to CDED, which normalizes exposure to hospital stay length.

Spearman’s rank correlation (rₛ) assessed the relationship between the number of drug administrations at admission or discharge and CDE or CDED. A strong correlation (rₛ close to 1 or -1) suggests that the number of administrations at a single time point is predictive of cumulative exposure

The difference in median CDE or CDED values between exposed (1) and non-exposed (0) patients provides a complementary perspective on the extent to which conventional, binary time-point metrics reflect overall cumulative drug burden.
