# Supplementary material for: Methodological Development and Assessing Prescribing Determinants Through Cumulative Drug Exposure in Hospitalized Patients: Proof-of-Concept Retrospective Study
Source: JMIR Med Inform. 2026 Apr 16;14:e76961. doi: 10.2196/76961 (PMC13086184; doi:10.2196/76961)
Supplement: Multimedia Appendix 3 [file medinform-v14-e76961-s003.docx]

## Supplementary datas :

|  | **Dimension 1** | | **Dimension 2** | | **Dimension 3** | |
| --- | --- | --- | --- | --- | --- | --- |
|  | *cos²* | *Contribution*  *(%)* | *cos²* | *Contribution*  *(%)* | *cos²* | *Contribution*  *(%)* |
|  |  |  |  |  |  |  |
| **Number of drug administration at admission** | 0.33 | 7.96 | 0.54 | 17.54 | 0.001 | 0.01 |
| **Number of drug administration at discharge** | 0.45 | 10.91 | 0.15 | 4.85 | 0.19 | 11.18 |
| **Number of drug interaction at admission** | 0.23 | 5.69 | 0.57 | 18.51 | 0.02 | 1.25 |
| **Number of drug interaction at discharge** | 0.53 | 12.85 | 0.04 | 1.52 | 0.09 | 5.32 |
| **Presence of PP at admission** | 0.34 | 4.31 | 0.60 | 13.97 | 0.001 | < 0.001 |
| **Presence of PP at discharge** | 0.62 | 3.89 | 0.19 | 2.19 | 0.11 | 3.72 |
| **Presence of HPP at admission** | 0.44 | 4.45 | 0.20 | 3.80 | 0.01 | 1.16 |
| **Presence of HPP at discharge** | 0.29 | 2.52 | 0.03 | 0.51 | 0.11 | 13.06 |
| **Presence of DDI at admission** | 0.27 | 2.18 | 0.60 | 8.85 | 0.001 | 0.43 |
| **Presence of DDI at discharge** | 0.61 | 4.24 | 0.27 | 2.65 | 0.001 | 3.10 |
|  |  |  |  |  |  |  |
| **CDE to PP** | 0.23 | 5.59 | 0.17 | 5.82 | 0.33 | 18.90 |
| **CDE to HPP** | 0.47 | 11.41 | 0.13 | 4.15 | 0.29 | 16.37 |
| **CDE to DDI** | 0.49 | 11.87 | 0.06 | 2.23 | 0.29 | 16.48 |
