# Supplementary material for: Methodological Development and Assessing Prescribing Determinants Through Cumulative Drug Exposure in Hospitalized Patients: Proof-of-Concept Retrospective Study
Source: JMIR Med Inform. 2026 Apr 16;14:e76961. doi: 10.2196/76961 (PMC13086184; doi:10.2196/76961)
Supplement: Multimedia Appendix 4 [file medinform-v14-e76961-s004.docx]

## Supplementary datas :

|  | **Dimension 1** | | **Dimension 2** | | **Dimension 3** | |
| --- | --- | --- | --- | --- | --- | --- |
|  | *cos²* | *Contribution (%)* | *cos²* | *Contribution*  *(%)* | *cos²* | *Contribution*  *(%)* |
|  |  |  |  |  |  |  |
| **Number of drug administration at admission** | 0.46 | 9.87 | 0.38 | 13.59 | 0.02 | 1.43 |
| **Number of drug administration at discharge** | 0.34 | 7.46 | 0.32 | 11.25 | 0.17 | 12.25 |
| **Number of drug interaction at admission** | 0.38 | 8.33 | 0.46 | 16.00 | 0.004 | 0.28 |
| **Number of drug interaction at discharge** | 0.48 | 10.55 | 0.16 | 5.61 | 0.009 | 0.66 |
| **Presence of PP at admission** | 0.52 | 5.72 | 0.40 | 11.37 | 0.03 | 3.24 |
| **Presence of PP at discharge** | 0.53 | 2.83 | 0.36 | 5.00 | 0.02 | 1.32 |
| **Presence of HPP at admission** | 0.58 | 5.29 | 0.10 | 2.56 | 0.07 | 7.22 |
| **Presence of HPP at discharge** | 0.36 | 2.63 | 0.001 | 0.23 | 0.37 | 26.43 |
| **Presence of DDI at admission** | 0.43 | 2.86 | 0.40 | 6.86 | 0.07 | 4.93 |
| **Presence of DDI at discharge** | 0.54 | 3.15 | 0.38 | 5.72 | 0.03 | 0.10 |
|  |  |  |  |  |  |  |
| **CDED to PP** | 0.32 | 6.98 | 0.04 | 1.72 | 0.09 | 6.81 |
| **CDED to HPP** | 0.57 | 12.40 | 0.03 | 1.17 | 0.15 | 10.56 |
| **CDED to DDI** | 0.54 | 11.86 | 0.008 | 0.21 | 0.27 | 18.94 |
